# Preclinical Assessment of Low Doses of Cisplatin in the Management of Acute Promyelocytic Leukemia

**DOI:** 10.16966/2381-3318.113

**Published:** 2015-10-15

**Authors:** Shaloam R Dasari, Venkatramreddy Velma, Clement G Yedjou, Paul B Tchounwou

**Affiliations:** Cellomics and Toxicogenomics Research Laboratory, NIH/NIMHD RCMI-Center for Environmental Health, College of Science, Engineering and Technology, Jackson State University, 1400 Lynch Street, Box 18750, Jackson, MS 39217, USA

**Keywords:** Cisplatin, HL-60 cells, Cell viability, Oxidative stress, Oxidative damage

## Abstract

Cis-diamminedichloroplatinum (II) (cisplatin) is the most widely used chemotherapeutic drug for various cancers, but its effectiveness is limited by tumor cell resistance and the severe side effects it causes. Since high level of cisplatin is cytotoxic to both cancer and normal cells, the goal of the present study was to explore the effectiveness of prolonged low doses of cisplatin in the management of leukemia. To achieve our goal, human leukemia (HL-60) cells were treated with different doses (1, 2, or 3 µM) of cisplatin for 24, 48, 72 and 96 hours. Cell viability was assessed by MTS assay. Both oxidative stress damage and genotoxicity were estimated by antioxidants, lipid peroxidation, and comet assays, respectively. Data obtained from the MTS assay demonstrated that cisplatin treatment decreased the number of viable tumor cells by direct cell killing or by simply decreasing the rate of cellular proliferation in a dose- and time-dependent fashion. The results of the lipid peroxidation showed a significant increase (p<0.05) of malondialdehyde levels with increasing cisplatin doses. Results obtained from super oxide dismutase and catalase assays showed a gradual increase in antioxidant enzyme activity in cisplatin-treated cells compared to control cells. Data generated from the Comet assay demonstrated a significant dose-dependent increase in genotoxicity with respect to DNA damage as a result of cisplatin treatment. Taken together, our research demonstrated that cisplatin-induced cytotoxicity in HL-60 cells is mediated at least in part via induction of oxidative stress and oxidative damage.

## Introduction

Cisplatin, cis-diamminedichloroplatinum (II), is a well-known chemotherapeutic drug and is one of the most widely used drugs for the treatment of various cancers [1,2]. Cisplatin is clinically proven to combat different types of cancers including sarcomas, cancers of soft tissue, bones, muscles, and blood vessels. Recent developments in the chemotherapy of such cancers have yielded better prognosis and therefore have led to these diseases becoming less life threatening [3]. From the molecular perspective of cisplatin, it represents a perfect example of how a small alteration in chemical structure can significantly affect biological activity in target cells. Nine platinum analogs are currently in clinical trials around the world [4,5].

Cisplatin is known to bind cellular components such as DNA and proteins, and to form DNA and protein adducts as a result of cross links with DNA and protein molecules that hamper transcription and translation mechanisms [6,7]. Those DNA adducts impact cell cycle progression check points and determine whether the cell has to die or survive with the help of repair mechanisms [8]. In addition to the formation of DNA adducts, cisplatin induces oxidative stress, and modulates calcium signaling, and cell apoptosis [9,10]. It also regulates the expression of many proteins that play a major role in cell cycle regulation and signal transduction [8,11,12]. Cisplatin also mediates the mitogen activated protein kinases (MAPK) and jun amino-terminal kinase (JNK) pathways that coordinate various extra cellular signals to regulate cell growth, survival, and apoptosis [13,14].

Additionally, cisplatin induced oxidative stress may trigger cell death besides DNA damage. Oxidative stress is one of the important processes that induce toxicity by targeting the mitochondrion membrane potential, and eventually leads to the inhibition of calcium uptake due to loss of mitochondrial protein sulfhydryl group. The degree of oxidative stress induction is dependent on the exposure time and drug dose [15]. The thiol group (– SH) containing molecules play a major role by maintaining the intracellular redox homeostasis. Reactive oxygen species are generated when thiol radicals interact with molecular oxygen under induced conditions [3]. If the cells cannot control the generation of reactive oxygen species, they may eventually damage cellular membranes and trigger intrinsic and/or extrinsic pathways of apoptosis [16]. However, in contrary to entering the apoptotic pathway in some - patients, cells develop resistance to cisplatin due to a decrease in drug intake or a passively diffusion of drug [17,18].

Although some of the biochemical effects of cisplatin are well studied, the detailed mode of its potential therapeutic action at low doses has not yet been elucidated. The goal of present research was to assess the low dose effects and elucidate the cytotoxic mechanisms of cisplatin on HL-60 cells. Our results provide a scientific basis to identify the lowest dose of cisplatin that has a maximum impact in reducing cancer cell proliferation, thereby minimizing its side effects on normal/non-cancer cells. Hence, in this study, we have investigated the cytotoxicity, oxidative stress, lipid peroxidation and genotoxicity potentials of cisplatin at low levels of exposure to HL-60 cells.

## Methods and Materials

### Cell culture, chemicals and reagents

Cisplatin was obtained from University of Mississippi Medical Center (Jackson, MS). Iscove’s Modified Dulbecco’s Medium (IMDM) and HL-60 cells were purchased from American Type Culture Collection (ATCC) (Manassas, VA, USA). Fetal bovine serum (FBS), and penicillin – streptomycin were purchased from Sigma (St. Louis, MO, USA). IMDM contains 4 mM L-glutamine, 4500 mg/L glucose, and 1500 mg/L sodium bicarbonate, and is supplemented with 10% (v/v) FBS, and 1% (W/V) antibiotics. The live cells were incubated at 37°C in a 5% CO_2_ incubator (Thermoscientific, Waltham, MA, USA).

### Cell treatment

The experiments were conducted with three replicates for each control or treatment group. Uniform cell density of 1 × 10^6^ cells/mL was maintained for all the treatments and controls. Cells were treated with 1, 2, or 3 µM of cisplatin or left untreated, and incubated for various time periods (24, 48, 72, or 96 h) at 37°C in a humidified 5% CO_2_ incubator (Thermoscientific, Waltham, MA, USA).

### Cell proliferation assay

Cell viability was assessed as previously described [19] with CellTiter 96° AQueous One Solution Cell Proliferation Assay kit from Promega (Madison, WI, USA) with few modifications. Briefly, 100 µL aliquots of the treated or untreated cell suspension were seeded into 96-well polystyrene tissue culture plates and 20 µL of assay reagent was added to each well. After 60 min of incubation at 37°C, the absorbance was read at 490 nm with a 96 well plate reader from BMG LABTECH GmbH (Ortenberg, Germany).

### Lipid peroxidation assay

Malondialdehyde (MDA) concentrations were quantified as previously described [20] using lipid peroxidation assay kit (Abcam, Cambridge, MA, USA) with few modifications. After the each treatment, cell suspension was collected, centrifuged at 1,230 rpm for 5 min to get the cell pellet and the cell pellet was suspended in cell lysis buffer. After centrifugation at 13,000g for 10 min, a 200 µl aliquot was assayed for MDA levels according to the lipid peroxidation assay kit protocol. The absorbance of the sample was read at 532 nm with a 96-well plate reader from BMG Labtech GmbH (Ortenberg, Germany). The concentrations of MDA were determined from the standard curve.

### Superoxide dismutase (sod) and catalase assays

SOD and Catalase assays were carried out as previously described [21,22] with few modifications, using commercially available SOD assay kit and Catalase assay kit (Abcam, Cambridge, MA, USA), respectively. After each treatment, both the control and treated HL-60 cells were collected. Cell lysates were quantified for SOD and catalase activities according to the manufacturer’s protocol. The final absorbance for SOD was measured at 450 nm and catalase was measured at 570 nm using 96-well plate reader from BMG Labtech GmbH (Ortenberg, Germany).

### Single cell gel electrophoresis

Cisplatin genotoxicity in treated and untreated HL-60 cells was analyzed by alkaline single cell gel electrophoresis (Comet) assay as described earlier [23] with few modifications using Comet assay kit for single cell gel electrophoresis from Trevigen (Gaithersburg, MD, USA). All the precautions were taken to avoid the UV light effect on DNA. Low melting agarose was melted in boiling water and cooled down to 37°C. For each treatment, 75 µL of the agarose and cells mixtucre (ratio of 1:10) was placed on comet slides and solidified at 4°C. Cells were lysed with lysis solution for 30 min at 4°C, followed by denaturing the DNA with alkaline solution for 40 min at 37°C. The prepared slides were subjected to electrophoresis (1 volt/cm) for 10 min in TBE (Tris borate EDTA) buffer. After the electrophoresis, cells were fixed with 70% ethanol followed by staining with SYBR green. Epifluorescent microscope (Olympus BX51 TRF, USA) was used to observe the comet slides. The data was evaluated using the DNA damage analysis software (Loats Associates Inc., USA).

### Statistical analysis

Experiments were carried out in triplicates, and the data were presented as means ± SDs. To test for differences among and between experimental groups, one–way analysis of variance (ANOVA) and Student’s t-test were performed respectively, using SAS software available in the Biostatistics Core Laboratory available at the RCMI Center for Environmental Health at Jackson State University for testing differences. Data were considered statistically significant for p-values less than 0.05.

## Results

### Cisplatin inhibits HL60 cell proliferation

**Figure 1:**
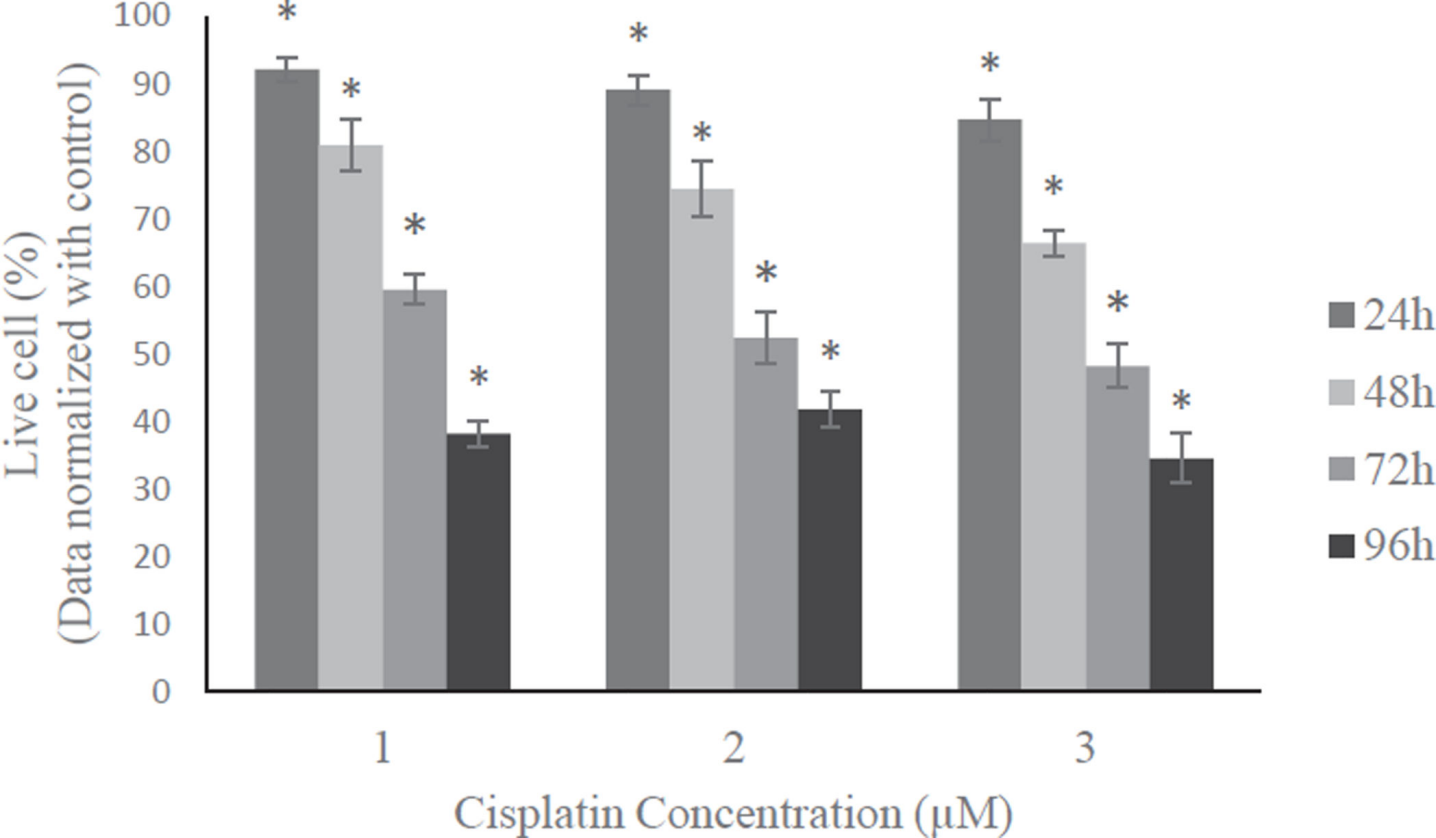
Cytotoxic effect of cisplatin on HL-60 cells. Cells were treated with 1, 2 and 3 µM of cisplatin for 24h, 48h, 72h and 96h. Cell viability was tested by cell proliferation assay using a spectrophotometer at 490 nm. Data was normalized with control to one. Results were expressed as means of three independent experiments ± standard deviations. p-values less than 0.05 were considered statistically significant.

Cell survival was measured in HL60 cells treated with 1, 2, or 3 µM cisplatin for 24, 48, 72, and 96 h incubation periods compared to the respective controls (Figure 1). The results indicate that the cell viability was decreased with increased cisplatin dose and incubation period. For 1 µM cisplatin, the percentages of cell viability were 92.0 ± 1.7%, 81.0 ± 3.8%, 59.0 ± 2.2% and 38.0 ± 1.9% for 24, 48, 72, and 96 h treatment, respectively. The recorded data for 2 µM cisplatin were 89.0 ± 2.2%, 74.0 ± 4.0%, 52.0 ± 3.9% and 41.0 ± 2.8% for 24, 48, 72, and 96 h treatment, respectively. The cell viability percentages for 3 µM cisplatin were 84.0 ± 3.1%, 66.0 ± 1.8%, 48.0 ± 3.3%, and 34.0 ± 3.6% for 24, 48, 72, and 96h treatment, respectively. Compared to the 1 µM treatment; cell survival rates decreased by about 8%, 15%, 11%, and 4% in 3 µM cisplatin, for 24, 48, 72, and 96h treatment, respectively. Overall, significant dose- and time-dependent decreases (p<0.05) were observed in cisplatin-treat cells compared to control cells.

### Cisplatin elevates SOD and catalase activities in HL60 cells

**Figure 2:**
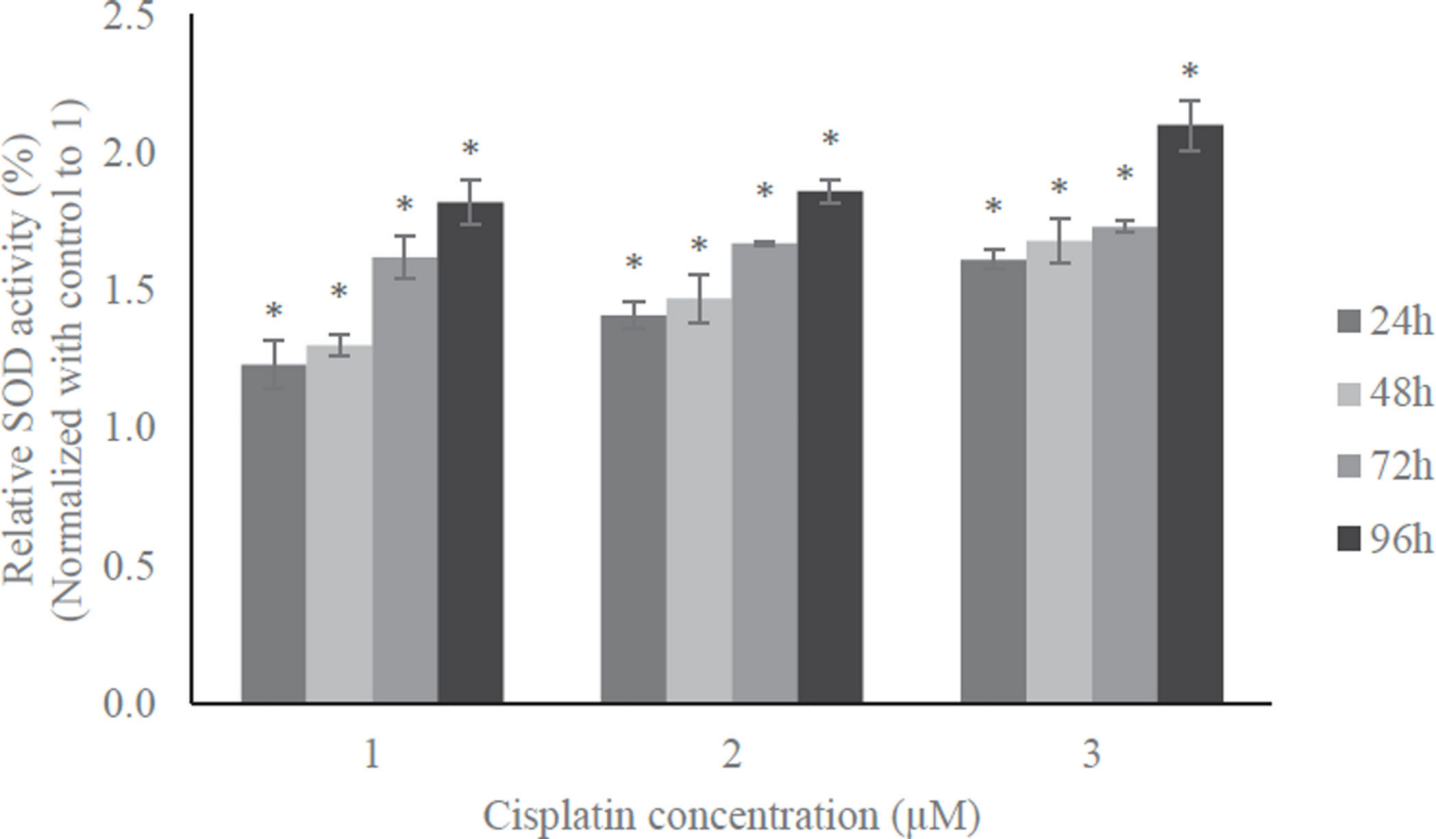
Effect of cisplatin on SOD activity in HL-60 cells. SOD activity was measured for the HL-60 cells challenged with 1, 2 and 3 µM of cisplatin for 24h, 48h, 72h and 96h. Post treatment, cells were lysed and SOD activity was quantified. The absorbance of each sample was measured at 450 nm and results were presented in the graph. Data was normalized with control to 1 and expressed as means of three independent experiments ± standard deviations. p-values less than 0.05 were considered statistically significant.

**Figure 3:**
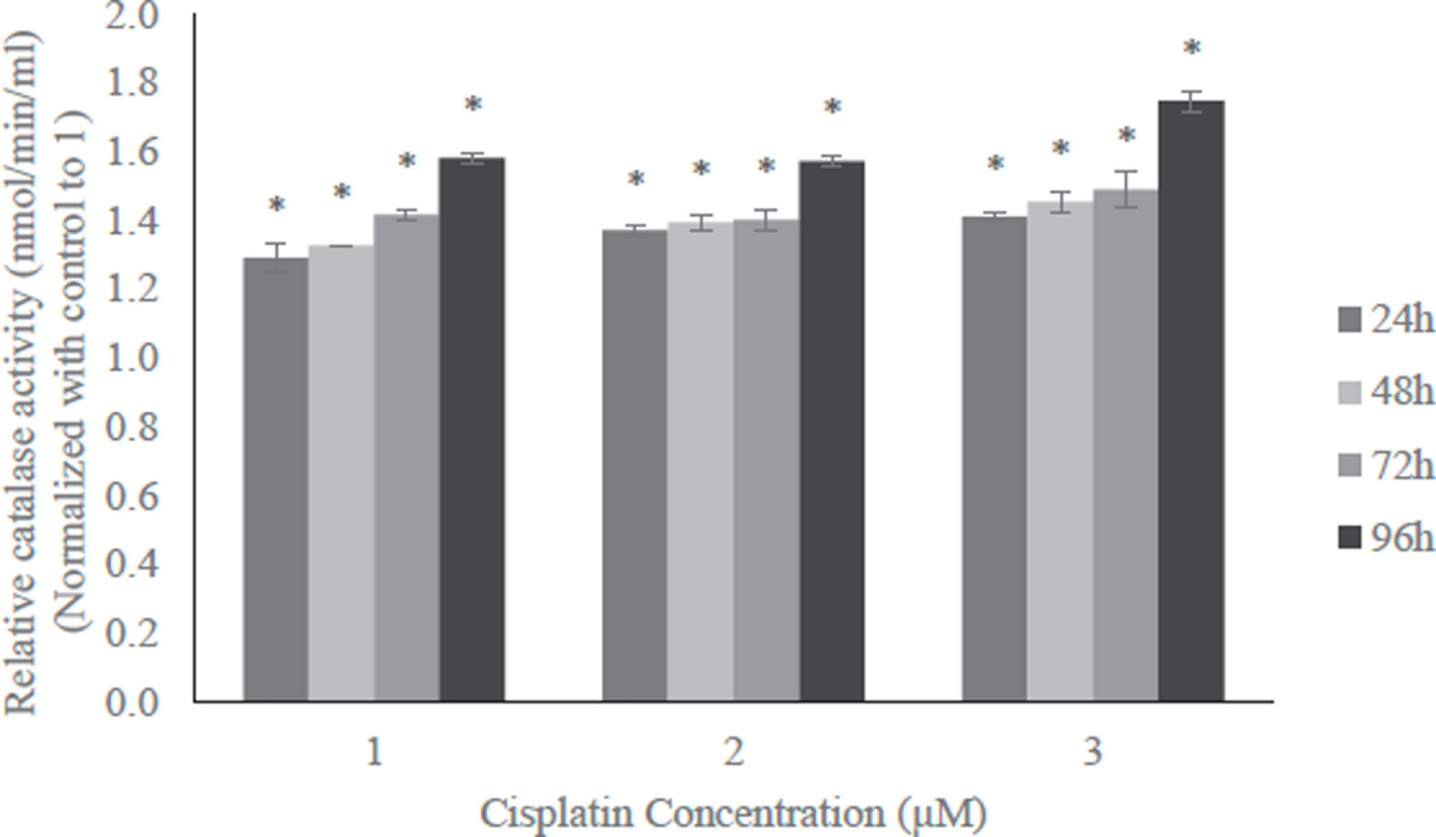
Effect of cisplatin on catalase activity in HL-60 cells. HL-60 cells were incubated with 1, 2, and 3 µM of cisplatin for 24h, 48h, 72h, and 96h. After each treatment, catalase activity was estimated using spectrophotometer at 570 nm. Results were expressed in nmol/min/ml. Data was normalized with control to 1. Data were expressed as mean of three independent experiments ± standard deviations. p-values less than 0.05 were considered statistically significant.

SOD and catalase activities were estimated in untreated or treated HL60 cells with 1, 2, or 3 µM cisplatin for 24, 48, 72, and 96 h time periods. The results were presented in Figures 2 and 3 for SOD and catalase, respectively. SOD and catalase activities were increased as the cisplatin dose or time period increases. At the lowest concentration, 1 µM cisplatin, SOD activity was increased by 23%, 30%, 62%, and 82%, while catalase activity was increased by 29%, 33%, 41%, and 58% compared to their respective controls at 24, 48, 72, and 96 h of exposure, respectively. In 2 µM cisplatin-treated cells, SOD activity was increased by 41%, 47%, 67%, and 86% while catalase activity was increased by 37%, 39%, 40%, and 57% compared to their respective controls, at 24, 48, 72, and 96 h of exposure, respectively. At the highest concentration, 3 µM cisplatin, SOD activity was increased by 61%, 68%, 73%, and 110% while catalase activity was increased by 41%, 45%, 49%, and 75% compared to their respective controls at 24, 48, 72, and 96h of exposure, respectively. The increases in SOD and catalase activities in cisplatin-treated cells were significantly different from the respective controls (p<0.05).

### Cisplatin induces lipid peroxidation in HL60 cells

**Figure 4:**
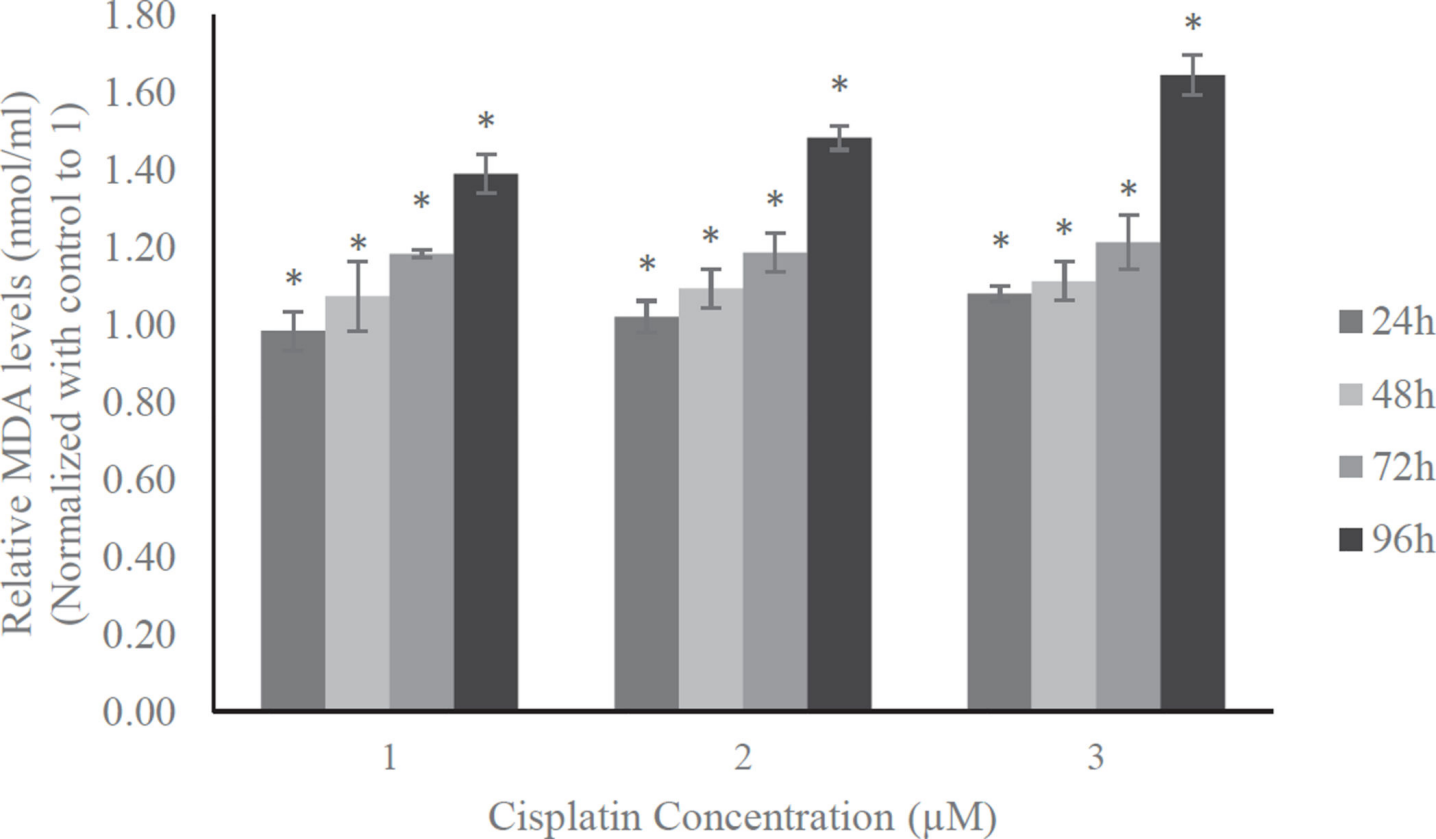
Effect of cisplatin on lipid peroxidation in HL-60 cells. HL-60 cells were treated with different doses of cisplatin or left untreated for 24h, 48h, 72h, and 96h. MDA levels were estimated in the treated and untreated cells. The amount of MDA (nmol/ml) was measured at 532 nm. Data was normalized with control to 1. The results were expressed as mean of three independent experiments ± standard deviations. p-values less than 0.05 were considered statistically significant.

One of the best methods for assessing oxidative stress is the estimation of MDA, a byproduct of lipid peroxidation. The MDA concentrations were estimated in HL-60 cells treated with cisplatin. The data are presented in Figure 4. As shown on this figure, the estimated MDA levels gradually increased in a dose- and time- response manner. After 96 h of exposure HL-60 cells treated with 1, 2 and 3 µM cisplatin induced 39, 48, and 64% more MDA compared to the respective controls. A similar trend was also found for any other exposure time periods. Hence, the increased MDA levels for all the tested concentrations and time periods were significantly higher than the respective controls (p<0.05).

### Cisplatin induces DNA damage in HL60 cells

**Figure 5:**
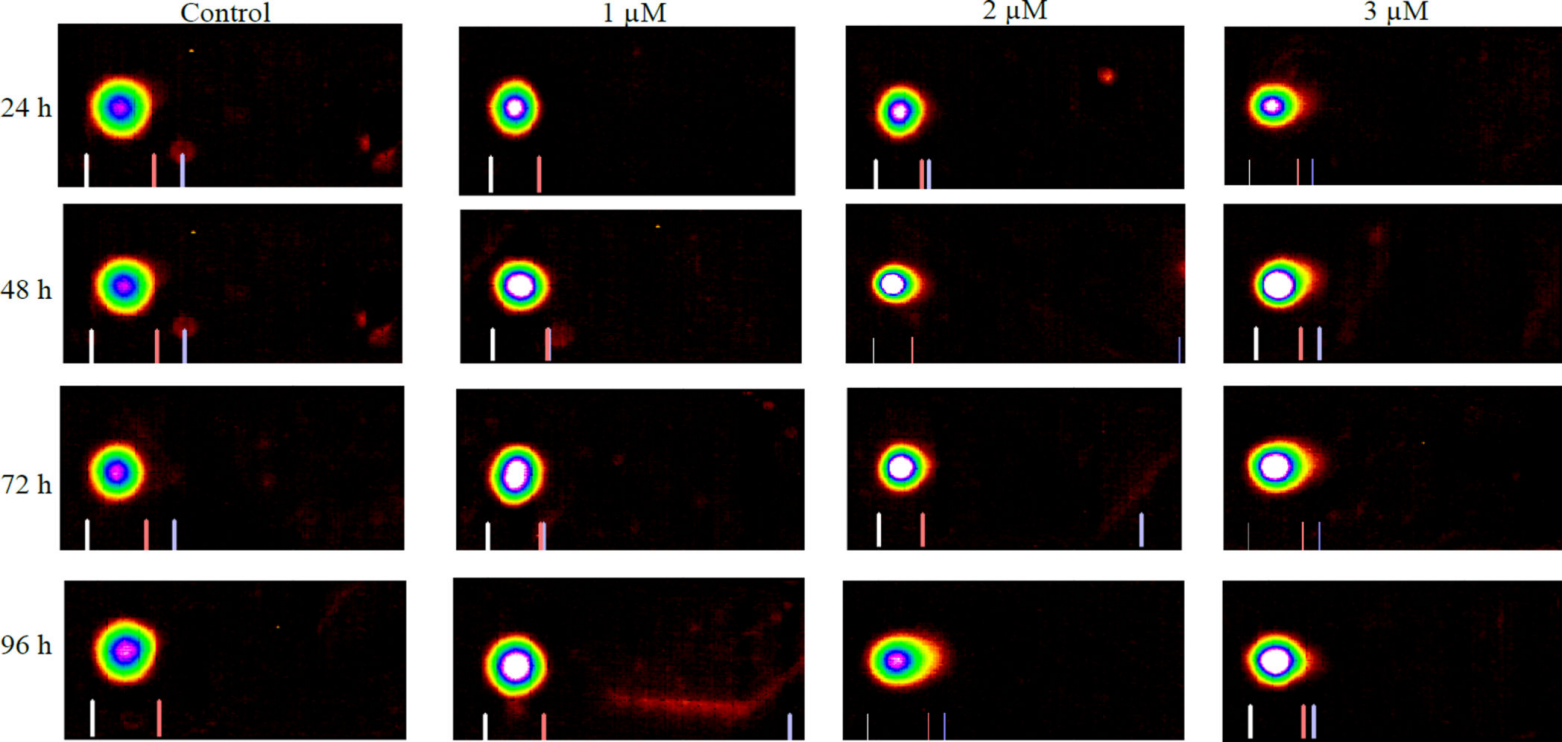
Representative Comet assay images of HL-60 cells exposed to cisplatin 0, 1, 2, or 3 µM for 24, 48, 72, and 96 h. The treated and untreated cells were subjected to single cell gel electrophoresis, stained with SBGR, and analyzed as described in the Materials and Methods section.

**Figure 6:**
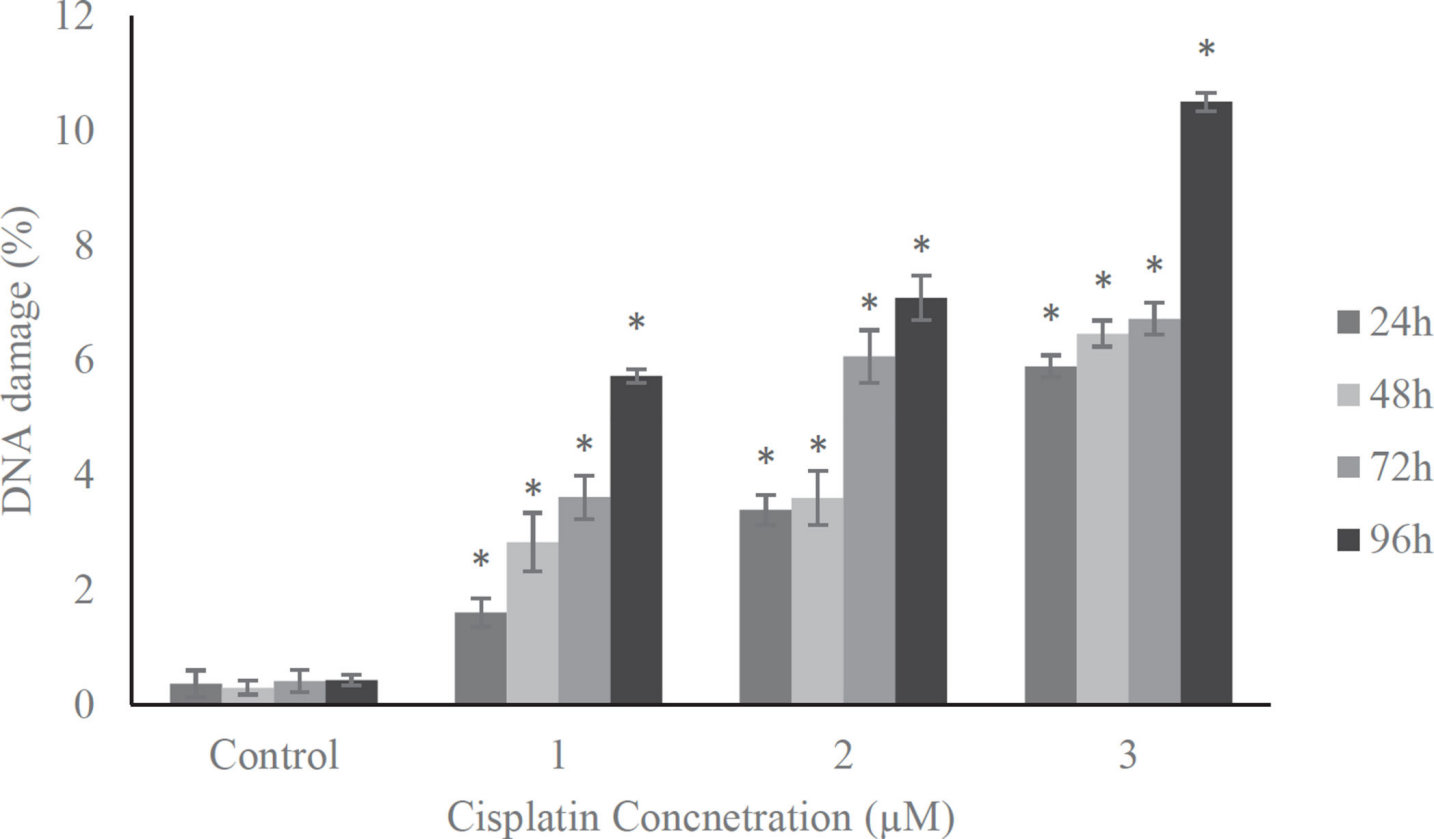
DNA damage in HL-60 cells treated with 0, 1, 2, or 3 µM cisplatin for 24, 48, 72, and 96 h. Data represent the percentages of DNA damage expressed as means of three independent experiments ± standard deviations. p-values less than 0.05 were considered statistically significant.

Treatment of HL60 cells with cisplatin at doses 1, 2 or 3 µM for a period of 24, 48, 72, and 96 hour shows dose- and time-response relationships with regard to DNA damage. Figure 5 presents the representative Comet assay images of HL-60 cells showing substantial increases in DNA damage at higher levels of cisplatin exposure. These qualitative data were analyzed quantitatively based on three replicates using the DNA damage analysis software (Loats Associates Inc., USA), The quantified DNA damage data presented in Figure 6 indicated that HL-60 cells treated with 3 µM cisplatin for 96 h induced the highest level of DNA damage, whereas those treated with 1 µM cisplatin for 24 h induced lowest level of DNA damage. At 1 µM, the percentages of DNA damage were1.6 ± 0.12%, 2.8 ± 0.51%, 3.6 ± 0.47%, and 5.7 ± 0.23% for 24, 48, 72, and 96 h exposure, respectively. At 2 µM these percentages were 3.4 ± 0.19%, 3.60 ± 0.38%, 6.1 ± 0.46% and 7.1 ± 0.28% for 24, 48, 72, and 96 h exposure, respectively. At the highest dose, 3 µM of cisplatin, the percentages of DNA damage were 5.9 ± 0.09%, 6.5 ± 0.12%, 6.7 ± 0.39%, and 10.5 ± 0.16% for 24, 48, 72, and 96 h exposure, respectively. The induced DNA damage is significantly higher for all the tested concentrations compared to the respective controls (p<0.05).

## Discussion

The present study was carried out to investigate the cytotoxicity, oxidative stress, and genotoxicity of cisplatin in HL60 cells. The main aim of this study was to research the anti-cancer potential of low doses of cisplatin, in order to minimize effect on non-cancer cells. We evaluated cell viability for cytotoxicity, anti-oxidants levels and lipid peroxidation for oxidative stress, and comet assay for genotoxicity.

Cisplatin is a neutral inorganic chemical compound that induces cytotoxicity by characteristic inhibition of cell cycle progression as a result of DNA adducts formation and subsequent induction of apoptosis. To exhibit toxicity, cisplatin has to hydrolyze with water molecules to become an active molecule that interacts with various macro molecules in the cell [17,24].The reactive cisplatin activity is dependent on the endogenous nucleophiles including glutathione, methionine, metallothionein, and other cellular components [2,25]. The endogenous nucleophiles act as a defense mechanism to counter attack cisplatin induced toxicity at lower levels of exposure.

In this study, HL60 cells were treated with 1, 2, or 3 µM cisplatin for various time periods. Study results show a significant cytotoxic effect in all tested doses (Figure 1). The lowest tested dose, 1 µM cisplatin, inhibited almost 60% of cell proliferation compared to the controls. The anti-proliferative or anti- cancer properties of cisplatin are in agreement with the previous reports that cisplatin inhibited cell proliferation rate in various cancers [26–28].

The eventual inhibition of cell proliferation or cytotoxicity in cells could be the result of collective mechanisms of cisplatin-induced oxidative stress, genotoxicity and other cellular responses. Hence, we determined whether oxidative stress plays a key role in cisplatin-induced toxicity in HL60 cells. We found that the activities of antioxidant enzymes such as SOD and catalase significantly increased in cisplatin-treated cells compared to control cells. These increases in enzymatic activities were both dose- and time-dependent (Figures 2 and 3).

As a part of defense mechanism, SOD catalyzes the dismutation of superoxide anion to hydrogen peroxide (H_2_O_2_) and the H_2_O_2_ become the substrate for the catalase. The induction of these two enzymes in the present study can be considered as an adaptive mechanism to combat the reactive oxygen species induced by cisplatin exposure. The dose-and time-dependent increases in antioxidant levels in the present study are consistent with the previous reports on cisplatin-induced reactive oxygen formation in a concentration- and time-dependent manner [15]. As a result of elevated antioxidants in response to oxidative stress, we observed a significant induction of MDA, alipid peroxidation byproduct, in HL60 cells treated with cisplatin (Figure 4). It has been previously reported that the release lipid peroxidation products increases carbonylation of proteins, induces oxidative damage of cell membranes, and those events may lead to the initiation of cell death [29–33].

In addition to oxidative stress, cisplatin induces genotoxocity and it is probably the most effective way to inhibit cell proliferation or cell cycle progression. Reactive cisplatin has been reported to react with functional residues such as sulfhydryl groups in proteins and DNA, specifically, nucleophilic sites of purines in DNA, leading to the formation of DNA-protein and DNA-DNA inter strand or intra strand cross links [34–37]. In the present study, the reported DNA damage was dose- and time-dependent (Figures 5 and 6). For 96 h treatment, DNA damage reported for 1 µM is almost 5% whereas for 3 µM is almost 10% higher than the respective controls (Figure 6). These findings are in agreement with cisplatin induced oxidative stress (Figures 2 and 3) and lipid peroxidation (Figure 4) in a dose-and time-dependent fashion. The induced DNA damage could be result of inter and intra strand cross links or culmination of both oxidative stress and DNA cross links. The current findings are consistent with previous reports that cisplatin interacts with purine bases of DNA and form DNA adducts to induce toxicity [8,38].

Cisplatin has also been reported to induce apoptosis and changes in cell cycle progression in wild type p53 and p53 deleted hepatoma cell lines [39]. The HL60 cells lack p53 protein, while cells with wild type p53 have been reported to show more sensitivity to cisplatin compared to p53-deficient cells [40]. These studies suggest that p53 activation could sensitize the cells to cisplatin toxicity. It has been reported that cisplatin induces apoptosis through both p53 dependent and independent pathways [39,41,42]. In consistence with the results of our study, it has been pointed out that cisplatin inhibits cell proliferation irreversibly by arresting the cells in G1 phase of cell cycle [43]. However, additional studies are required to elucidate the molecular mechanisms of cisplatin-induced growth inhibition, oxidative stress, cell cycle modulation, and genotoxicity in cancer cells.

## Conclusion

In this study, we investigated cisplatin-induced cytotoxicity, oxidative stress and genotoxicity at low levels of exposure (1, 2, and 3 µM) and at various time periods (24, 48, 72 and 96h). Cisplatin significantly inhibited cell proliferation, and induced antioxidants levels and lipid peroxidation even at lowest dose of 1 µM. Also, cisplatin significantly induced DNA damage in HL60 cells at all treatment doses. Taken together, low doses of cisplatin show a significant activity against HL-60 cells, and hence, may be used in combination with arsenic trioxide (ATO) to improve treatment and reduce potential side effects in acute promyelocytic leukemia patients. However, additional research is required to study the combined effect of cisplatin and ATO in order to determine their potential for use in APL chemotherapy.
